# Histological and Molecular Evaluation of HPV in Primary Tumors and Lymph Node Metastases of Penile Cancer

**DOI:** 10.3390/cancers18091350

**Published:** 2026-04-23

**Authors:** Luiza Dorofte, Sabina Davidsson, Jessica Carlsson, Mats G. Karlsson, Gabriella Lillsunde Larsson

**Affiliations:** 1 Department of Laboratory Medicine, Faculty of Medicine and Health, Örebro University, 701 85 Örebro, Sweden; 2 Department of Urology, Faculty of Medicine and Health, Örebro University, 701 85 Örebro, Sweden; 3 School of Health Sciences, Örebro University, 701 82 Örebro, Sweden

**Keywords:** penile cancer, HPV genotypes, p53, p16

## Abstract

Background: Penile squamous cell carcinoma is an uncommon neoplasm that arises via two carcinogenic pathways, one linked to HPV infection and the other to chronic inflammation and p53 alterations. Objective/Methods: We assessed the distribution of HPV genotypes in penile tumors and subsequent inguinal metastases in a cohort of 343 patients, analyzing their concordance with p16 stain and histological subtype, as well as the predictive significance of HPV tumor status and the immunohistochemical expression of p16 and p53 in inguinal lymph node metastasis (ILNM). Results: The overall prevalence of HPV in primary penile tumors was 42.9%, with high-risk HPV genotypes detected in 95.2% of HPV-positive cases. HPV16 was the most prevalent genotype identified in both primary tumors and metastases. However, other genotypes, including the low-risk HPV82, were also found in metastatic disease. We observed good concordance between HPV tumor status and histological subtype and very good concordance between HPV tumor status and p16 staining, with Cohen’s kappa (κ) values of 0.80 (*p*-value < 0.001) and 0.83 (*p* < 0.001), respectively. Conclusions: In most HPV-positive metastatic cases, the same HPV genotypes were detected in both the metastasis and the penile tumor. Both p16 staining and histological subtype can serve as surrogates for molecular HPV testing in lower resourced settings. In this dataset, no significant association was found between HPV status, p16 expression, or p53 expression and the presence of ILNM, suggesting their limited utility as predictive markers for ILNM in penile cancer.

## Simple Summary

Penile cancer is a rare type of tumor, with about half of all cases associated with HPV infection. In penile cancer patients, the status of the lymph nodes is the most important prognostic factor for survival. Sentinel node surgery for the identification of micrometastasis is not recommended in all patients due to the risk of complications. The aim of this study was to determine the distribution of HPV in penile tumors and lymph node metastases, as well as whether immunohistochemical markers (p16 and p53) and HPV tumor status can be used to predict lymph node metastasis. The same HPV genotypes were found in both the metastasis and the penile tumor in most HPV-positive metastatic cases. No significant association was found between HPV status, p16 expression, or p53 expression and the presence of lymph node metastases.

## 1. Introduction

As a rare form of malignant tumor, penile cancer is mostly seen in low- and middle-income countries, with the highest incidence rates of up to 6.8 per 100,000 men reported in countries in Africa, Asia, and South America [1]. Even though the incidence in the United States and high-income European countries is much lower, ranging from 0.1 to 1 per 100,000 men [2], there has been an increase in age-related incidence in many of these countries [3]. In Sweden, the incidence of penile cancer is currently 2.6 per 100,000 men [4], and an increase in the age-related incidence has been noted in all Nordic countries, where it now exceeds 2.0 per 100,000 men [5].

There are two known pathways involved in penile cancer carcinogenesis. One is associated with chronic inflammatory conditions (most commonly lichen sclerosus), while the other is related to human papillomavirus (HPV) infection [6]. Approximately 40–50% of penile cancers are HPV-related [7,8], most often involving the high-risk genotypes 16 and 18 [7,9,10]. The most recent World Health Organization (WHO) classification of penile cancer recommends that penile cancer be classified into HPV-associated and HPV-independent histological subtypes. While up to two-thirds of cases can be classified based on morphology alone, immunohistochemical staining (IHC) for p16 and/or molecular methods are recommended in difficult cases [11].

Metastatic penile cancer initially spreads to the inguinal lymph nodes and then the pelvic lymph nodes. The most important factor associated with patient survival is the presence of inguinal lymph node metastasis (ILNM) [12,13]. In patients with clinically negative inguinal lymph nodes and histologically proven metastasis on cytology or dynamic sentinel node biopsy (DSNB), early inguinal lymph node dissection is associated with a much higher survival rate than delayed lymph node dissection [14].

The current guidelines do not recommend prophylactic DSNB in all patients with clinically negative lymph nodes, due to the associated complication rate [15,16]. As 25% of patients with clinically negative lymph nodes already have nodal micrometastasis at the moment of diagnosis [17], predictive factors for metastatic disease are needed to identify patients who would benefit from inguinal surgery. Current imaging modalities lack sufficient sensitivity to detect occult inguinal lymph node metastases (ILNM), and no validated predictive biomarkers are available. Consequently, clinically node-negative patients are stratified into risk groups based on the histopathological characteristics of the primary tumor. According to the current risk grading based on histological grade and pT stage, patients with clinically negative lymph nodes and low-risk (pT1a, G1) penile tumors are monitored, whereas those assessed as intermediate and high-risk for metastatic disease (pT1aG2 and pT1b) receive DSNB [18] as part of standard treatment. According to the recommended risk grading, only a low number of patients are included in the low-risk group, resulting in a high number of patients who receive inguinal lymph node surgery, which is associated with an increased risk of morbidity and complications. Therefore, there is an unmet clinical need for sensitive and accessible biomarkers that enable the early detection of ILNM.

The relationship between HPV-associated malignant tumors and patient survival has been well documented. For example, in vulvar cancer or oropharyngeal SCC, HPV-related tumors have been associated with better survival [19,20]. In patients with penile cancer, patients with HPV-associated tumors have shown better survival than patients with HPV-independent tumors. However, to date, HPV tumor status has shown no prognostic value in the prediction of ILNM [21]. A thorough evaluation of HPV in both primary penile tumors and corresponding lymph node metastases is required to further understand HPV’s role in disease progression and potentially identify any association with ILNM.

Although the mechanisms for HPV-independent carcinogenesis in penile cancer are less understood, they appear to be linked to TP53 mutations [22,23]. Recent studies have shown that penile tumors with abnormal p53 IHC are more aggressive and associated with a higher risk for ILNM and poor prognosis [24,25]. However, additional larger studies are needed to better evaluate both HPV and p53 as biomarkers for penile cancer progression.

The aim of the present study was to determine the distribution of HPV in primary and metastatic disease and its concordance with p16 and histological subtype, as well as the potential predictive value of HPV tumor status and immunohistochemical expression of p16 and p53 for ILNM in patients with penile cancer.

## 2. Materials and Methods

Tumor material from 356 consecutive patients who underwent surgical treatment for penile cancer from 2009 to 2018 in Örebro University Hospital, Örebro, Sweden, was retrieved from the local biobank. After the exclusion of cases with insufficient tumor material (*n* = 13), the final cohort included material from 343 patients. The lymph node status was histologically assessed in a total of 305 patients (in 302 patients on either DSNB or lymph node dissection and in 3 patients with cytologically confirmed metastasis). Lymph node metastasis was present in 102 patients at the time of diagnosis (Table 1).

Paraffin blocks containing representative material from the invasive penile tumor and corresponding lymph node metastasis were selected by the pathologist (LD).

The tumor histological subtype, histological grade, and tumor stage were assessed on hematoxylin and eosin-stained slides. The pathologist was blinded to HPV tumor status during the morphological evaluation.

### 2.1. Assessment of Histological Subtype

The histological grade and subtype classification were assessed in accordance with the WHO and ISUP guidelines [26,27]. Penile squamous cell carcinoma is grouped into HPV-associated and HPV-independent histological subtypes. The HPV-independent category includes the usual subtype (including pseudohyperplastic and pseudoglandular patterns), verrucous and its variant carcinoma cuniculatum, the papillary subtype, and the sarcomatoid subtype. The HPV-associated category includes the basaloid subtype, warty carcinoma, clear cell carcinoma, and lymphoepithelioma-like carcinoma. Mixed histological subtypes, predominantly from the same group, are observed in up to 25% of penile cancer cases [27].

### 2.2. HPV Analysis

For HPV analysis, whole sections of selected tissue blocks (2–5, 10 μm) or parts of sections scraped from tissue slides were used for DNA extraction with the QIAamp DNA mini kit (Qiagen GmbH, Hilden, Germany), following the manufacturer’s instructions. Whole sections were used on paraffin blocks from the metastasis and from the penile tumor when only the invasive component was present in the tissue block. In cases where both invasive and PeIN components were present in the same block, a representative area from the invasive component was scraped for the analysis.

HPV genotyping was performed using Anyplex™ II HPV28 (Seegene, Seoul, Republic of Korea), a real-time PCR method capable of detecting 28 HPV genotypes (HPV6, 11, 16, 18, 26, 31, 33, 35, 39, 40, 42, 43, 44, 45, 51, 52, 53, 54, 56, 58, 59, 61, 66, 68, 69, 70, 73, and 82) as well as the human Beta-globin (HBB) gene. This method has previously been evaluated on archived formalin-fixed paraffin-embedded material by Lillsunde et al., and was shown to be highly sensitive for these types of samples [28].

HPV16, 18, 31, 33, 35, 39, 45, 51, 52, 56, 58, and 59 were considered high-risk genotypes.

### 2.3. Immunohistochemical Staining of p16 and p53

Tissue microarrays (TMAs) comprising representative samples from the invasive tumor component were constructed by using 3D HISTECH TMA Grand Master (Danjier Company, Budapest, Hungary). Three cores of 0.6 mm from the primary tumor were obtained from each patient when available. While no specific validation for immunohistochemical staining on TMA has been performed in penile cancer, TMAs are a reliable platform for immunohistochemical analysis, with multiple studies demonstrating high concordance between TMA cores and whole-tissue sections. This has been validated in gynecologic squamous malignancies and is widely extrapolated to penile squamous cell carcinoma [29,30]. TMA sections (4 µm) were subjected to immunohistochemistry staining for p53 (DAKO GA616 (DO-7)) and p16 (Ventana 805-4713/825-4713 (E6H4)) using the Autostainer Link 48 platform (Agilent Dako, Santa Clara, CA, USA).

### 2.4. Evaluation of p16 and p53 Staining

p16 staining was defined as positive when there was block staining in the invasive epithelial nests with continuous strong nuclear or combined nuclear and cytoplasmic staining of the basal layer, extending through at least one-third of the epithelial thickness according to the Lower Anogenital Squamous Terminology (LAST) recommendations [31].

p53 staining was considered abnormal if any of the following patterns were observed: strong nuclear staining of the basal layer, uniform strong nuclear staining in the whole epithelium, cytoplasmic staining with or without nuclear staining, or complete absence of nuclear staining. Heterogeneous nuclear staining or parabasal staining with a negative basal layer consistent with the wild-type pattern was considered normal. Basal cells were regarded as peripheral tumor cells next to the stroma in the invasive nests of squamous epithelium [32,33].

### 2.5. Statistical Analysis

Categorical variables are summarized using absolute and relative frequencies. The concordance between p16 immunohistochemical staining and HPV status was evaluated using Cohen’s kappa (Κ). The level of concordance was interpreted as poor (Κ = 0.00–0.20), fair (Κ = 0.21–0.40), moderate (Κ = 0.41–0.60), good (Κ = 0.61–0.80), or very good (Κ = 0.81–1.00) [34].

Only cases with documented lymph node staging or positive cytology consistent with metastatic disease were eligible for inclusion in the association analyses of lymph node metastasis (*n* = 305). Associations between metastatic disease and HPV status, p16, or p53 expression were analyzed using Chi-square tests or Fisher’s exact tests, as appropriate. Effect sizes were quantified using Cramer’s V. To estimate the precision of the effect size, 95% confidence intervals (CIs) were obtained using a non-parametric bootstrap procedure with 1000 resamples. Two-sided *p*-values < 0.05 were considered statistically significant.

To assess the incremental value of HPV-related biomarkers, nested multivariable logistic regression models were constructed with ILNM as the dependent variable. The analysis was restricted to patients with tumors classified as >T1aG2. The base model included established clinicopathological variables: lymphovascular invasion, perineural invasion, tumor grade, and histological subtype. Due to sparse data and model instability in the initial analysis, the tumor grade was dichotomized (Grade 1–2 vs. Grade 3), and the histological subtype was simplified into HPV-associated versus HPV-independent categories to ensure model convergence. A second model was then fitted by adding the HPV status, p16 expression, and p53 expression to the base model. The model performance was compared using the likelihood ratio test. Odds ratios (ORs) with 95% CIs were calculated. Model calibration was evaluated using the Hosmer–Lemeshow goodness of fit test. All statistical analyses were performed using IBM SPSS Statistics, version 27 (IBM Corp., Armonk, NY, USA).

## 3. Results

Selected tumor characteristics of the cohort are presented in Table 1. Of the 343 cases included in the study, the majority (59.2%) were lymph node-negative, while 11.1% had not undergone lymph node staging (pNx). No differences in the presence of lymph node metastases were observed between HPV-positive and HPV-negative tumors (*p* = 0.83, Cramer’s V = 0.017, 95% CI 0.001–0.131). However, HPV-negative tumors were more frequently of lower grade compared to HPV-positive tumors (*p* < 0.001, Cramer’s V = 0.36, 95% CI 0.29–0.43).

### 3.1. Histological Subtype

All the histological subtypes of penile SCC included in the WHO classification were identified in our cohort. In 212 (63%) cases, we found HPV-independent subtypes, with the most prevalent being the usual subtype 44.3% (*n* = 152), followed by the verrucous subtype 6.7% (*n* = 23) and the papillary, sarcomatoid, and mixed HPV-independent subtypes 10.8% (*n* = 37). From the 131 (37%) tumors from the HPV-associated category, the most prevalent was the basaloid subtype 12.2% (*n* = 42), followed by the warty–basaloid subtype 7.3% (*n* = 25), warty 6.7% (*n* = 23), and other rare as well as mixed HPV-associated subtypes 12% (*n* = 41).

### 3.2. HPV Status

**Figure 1:**
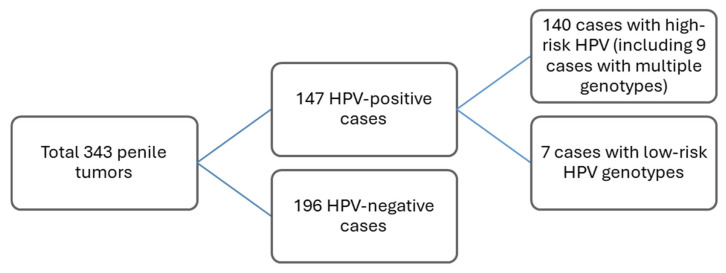
HPV status in primary tumors.

The overall HPV prevalence was 42.9% (147/343), based on HPV positivity in the invasive component. In the total cohort, 140 of the cases harbored a high-risk genotype (40.8%). Among the 140 cases, 125 (89.3%) presented with a single high-risk genotype or in combination with other genotypes (*n* = 15, 10.7%). Seven (2.0%) cases from the total cohort were positive for exclusively low-risk HPV genotypes: HPV42 (*n* = 3), HPV42 + HPV6 (*n* = 1), HPV82 (*n* = 1), HPV82 + HPV44 (*n* = 1), and HPV53 (*n* = 1) (Figure 1).

**Figure 2:**
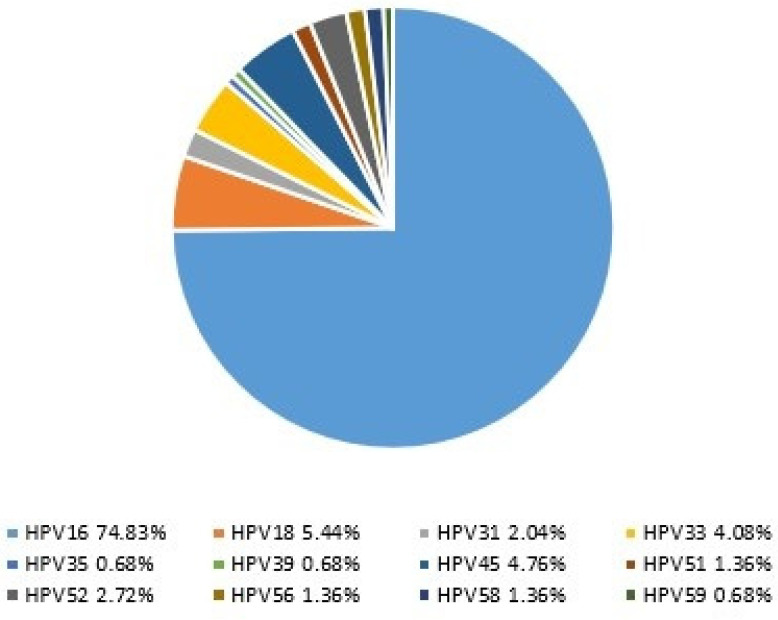
High-risk genotypes in the HPV-positive tumors.

Among the 140 cases with a high-risk genotype, the most common genotype result found was HPV16 in 78.6% (*n* = 110), followed by HPV18 5.7% (*n* = 8), HPV45 5.0% (*n* = 7), HPV33 4.2% (*n* = 6), HPV52 2.9% (*n* = 4), HPV31 2.1% (*n* = 3), HPV51 1.4% (*n* = 2), HPV56 1.4% (*n* = 2), HPV58 1.4% (*n* = 2), HPV35 0.7% (*n* = 1), HPV39 0.7% (*n* = 1), and HPV59 0.7% (*n* = 1) (Figure 2).

Out of 102 patients with confirmed metastatic disease, HPV analysis was performed on tissue from ILNM in 98 cases, whereas in four cases, the tumor tissue from metastasis was insufficient. Of the 102 patients, 46 (45.1%) had an HPV-positive primary tumor, while the remaining 56 (54.9%) were HPV-negative.

HPV16 was the most frequently detected genotype in HPV-positive cases that presented with metastasis (*n* = 41). Metastatic disease was also observed in patients with HPV18 (*n* = 1), HPV35 (*n* = 1), HPV45 (*n* = 1), HPV52 (*n* = 1), and HPV82 (*n* = 1).

**Figure 3:**
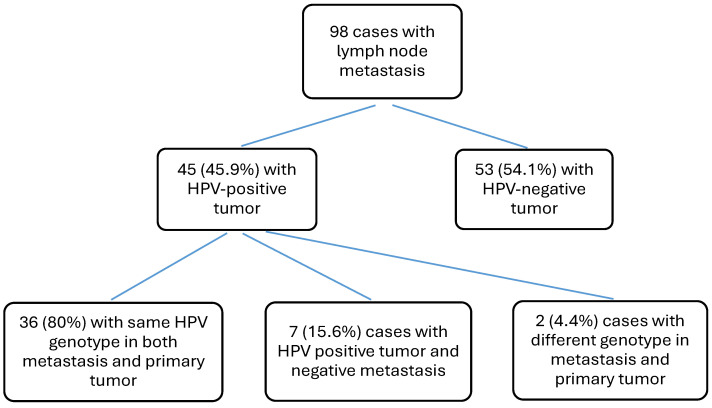
Overview of HPV findings in primary tumor and ILNM. Of the 102 cases with confirmed metastases, 98 cases had sufficient material for HPV analysis in both primary tumor tissue as well as in the metastasis.

In the group of HPV-positive tumors with ILNM, where HPV analysis could be performed on both components (*n* = 45), the same HPV was found in the metastasis and the primary tumor in 36 cases. In two cases, different genotypes were present in the metastasis and primary tumor, and in the remaining seven cases, the metastasis was HPV-negative (Figure 3 and Table 2).

### 3.3. Concordance for HPV Tumor Status and Histological Subtype of SCC

A good concordance was found between the histological subtype of squamous cell carcinoma and HPV tumor status (PCR result) with a Cohen’s kappa (κ) of 0.80 (*p*-value < 0.001), corresponding to 90.7% agreement. Of the 32 discordant cases, eight had HPV-associated histology but were HPV-negative. The remaining 24 cases had HPV-independent histology but were HPV-positive. From the seven cases where the tumor was positive for a low-risk HPV, five presented an HPV-associated histological subtype or mixed subtypes, and the other two showed a usual subtype of high grade.

When investigating whether any of the histological subtypes were more prone to metastasize, the usual, basaloid, warty, and warty–basaloid subtypes had very similar metastasis frequencies, ranging from 35.9% (basaloid) to 45.5% (warty-basaloid). None of the 17 patients with a verrucous subtype had ILNM, while mixed HPV-associated subtypes and mixed HPV-independent subtypes metastasized in 21.6% vs. 14.3% of the cases (Table 3).

### 3.4. Immunohistochemistry for p16 and p53

The immunohistochemical evaluation of p16 and p53 was successfully completed in 335 cases. In the remaining eight cases, adequate TMA material could not be obtained. A total of 131/335 (39.1%) tumors had positive block staining for p16, and 204/335 (60.9%) were negative cases. P53 was positive in 91/335 (27.2%) of the tested tumors, while the rest of the 244/335 (72.8%) cases were negative.

### 3.5. Concordance for p16 and HPV Status

Among the 131 tumors that were positive for p16, 122/131 (93.1%) were also positive for HPV infection. Of the 204 p16-negative tumors, 185/204 (90.6%) were HPV-negative. The results show a very good concordance between p16 immunohistochemical staining and HPV tumor status (PCR result) with a Cohen’s κ of 0.83 (*p* < 0.001). The overall agreement between p16 staining and HPV status was 91.6%. However, a subanalysis revealed that the agreement between p16 and HPV status (PCR result) was higher in high-risk HPV subtypes (88.8%) compared to low-risk HPV subtypes (42.9%).

### 3.6. Association Between HPV Tumor Status, p16 and p53 Staining, and ILNM

Among 305 cases with histologically confirmed lymph node status, the Chi-square test indicated that the HPV tumor status (PCR results) was not significantly associated with ILNM (*p*-value = 0.71, Cramer’s V = 0.026, 95% CI −0.083–0.135).

Histologically assessed lymph node status and IHC data were available in 298 cases. A total of 42/116 (36.2%) of the p16-positive cases, as well as 32/83 (38.6%) of the p53-positive cases, presented with lymph node metastasis. Chi-square analysis demonstrated that neither p16 positivity nor abnormal p53 staining was significantly associated with ILNM (*p* = 0.53 and *p* = 0.34, respectively), with corresponding small effect sizes (Cramer’s V = 0.04, 95% CI 0.003–0.15 for p16 and 0.06, 95% CI 0.003–0.18 for p53).

A sensitivity analysis restricted to high-risk HPV-positive tumors did not demonstrate any statistically significant association between HPV, p16, or p53 status and ILNM, supporting the overall findings.

To investigate whether the combination of HPV status (PCR result) and p16 staining was associated with ILNM, HPV + p16+ cases were compared to all other cases. In cases positive for both HPV and p16, 40/109 (36.7%) had metastatic disease while, in the remaining cases, 61/189 (32.3%) were metastasized. However, no significant association with ILNM status was evident (*p* = 0.45, Cramer’s V = 0.05, 95% CI 0.002–0.16).

A second subanalysis evaluated the association between p16 and p53 positivity and ILNM in patients with tumors ≥ pT1aG2 (*n* = 267). Among patients with ILNM, 42/101 (41.6%) had a p16-positive tumor, compared with 70/166 (42.2%) of patients without ILNM. P53 positivity was observed in 32/101 (31.7%) of patients with ILNM and 48/166 (28.9%) of those without ILNM. Neither p16 nor p53 positivity showed a statistically significant association with ILNM (*p* = 1.0 and *p* = 0.78, respectively), with small effect sizes (Cramer’s V = 0.006, 95% CI 0.002–0.133 for p16 and 0.029, 95% CI 0.002–0.152 for p53). HPV positivity was found among 44.6% (45/101) of patients with ILNM and among 46.4% (77/166) of ILNM-negative patients. No significant association was observed for HPV positivity (*p* = 0.8), with a small effect size (Cramer’s V = 0.08, 95% CI 0.003–0.139).

### 3.7. Multivariable Analysis

In the multivariable base model, lymphovascular invasion, perineural invasion, and grade 3 tumors were independently associated with ILNM. Lymphovascular invasion was associated with significantly increased odds of ILNM (OR 5.64, 95% CI 2.81–11.31), as was perineural invasion (OR 3.37, 95% CI 1.54–7.40). Tumors of grade 3 were also associated with higher odds of ILNM compared with grade 1–2 tumors (OR 2.77, 95% CI 1.37–5.58). In contrast, the HPV-associated histological subtype was not significantly associated with ILNM (OR 0.76, 95% CI 0.36–1.61).

The addition of HPV status, p16 expression, and p53 expression to the model did not result in a statistically significant improvement in the model fit (likelihood ratio test: χ^2^ = 5.27, df = 3, *p* = 0.15). Furthermore, none of the added biomarkers were independently associated with ILNM. Specifically, p16 positivity was not significant (OR 0.55, 95% CI 0.15–2.02), nor was p53 positivity (OR 0.56, 95% CI 0.24–1.32). HPV positivity also did not reach statistical significance (OR 3.42, 95% CI 0.87–13.47). Overall, these findings indicate that, for this dataset, the inclusion of HPV, p16, and p53 did not provide additional predictive value beyond established clinicopathological factors in the multivariable model.

In a stratified analysis of HPV-negative tumors, p53 expression remained non-significant in the multivariable analysis (OR 0.38, 95% CI 0.13–1.12). In the same subgroup, lymphovascular invasion (OR 4.55, 95% CI 1.65–12.51), perineural invasion (OR 4.97, 95% CI 1.73–14.27), and grade 3 (OR 4.43, 95% CI 1.47–13.39) remained independently associated with ILNM.

## 4. Discussion

Penile SCC is a rare type of tumor with a well-documented variation in geographic incidence, with rates in high-income European countries typically around 0.5–1 per 100,000 men, though higher in countries such as Lithuania and Latvia and lower in Netherlands and Switzerland. Parts of China report an incidence of 1–2 per 100,000 men and markedly higher incidence is found in northeast Brazil, where it may exceed 5–8 per 100,000 men [35]. These differences in reported incidence likely reflect variation in risk factors and socioeconomic conditions but can also be attributed to methodological differences in study design, registry quality, and standardization.

While less studied than the carcinogenesis of other anogenital tumors, the development of penile cancer is based on two pathways. HPV-induced carcinogenesis has been well documented, especially in cervical cancer, where HPV infection is responsible for over 99% of cases [29]. A systematic study by de Martel et al. in 2017 indicated that the prevalence of HPV in non-cervical cancers was 74% in vaginal cancer, 29% in vulvar cancer, 33% in penile cancer, and 25% in oropharyngeal cancer [36]. The HPV-independent pathway is less understood, but appears to be associated with TP53 mutations [22,37].

According to a meta-analysis published in 2019, the pooled prevalence of HPV in penile cancer is 50.8% (95% CI 44.8–56.7) [38]. In our cohort, the prevalence of HPV-positive SCC was 42.9%. The variation in prevalence in different studies can be explained by the study sample size, the distribution of HPV infection in the population, and socioeconomic factors, as well as methodological differences in the chosen assay.

According to a meta-analysis by Olesen et al., the most prevalent HPV genotype in penile SCC is HPV16 (68.3%), followed by HPV6 (8.1%) and HPV18 (6.9%) [38]. In our cohort, from the 147 HPV-positive cases, the predominant genotype identified was HPV16 (74.8%), followed by HPV18 (5.4%). HPV16 was the most predominant genotype in metastatic cases, corroborating findings from other studies. Given that most HPV-positive tumors in our study were HPV16-positive, HPV16 was predictably the predominant genotype found in metastasis. HPV6 was found as a coinfection with a high-risk HPV in 2% of cases.

While most HPV-associated SCCs are caused by high-risk genotypes, there are documented cases of low-risk HPV genotypes involved in the development of SCC, especially low-risk HPV6 and HPV11 [39]. These low-risk HPV genotypes have been demonstrated in cases of laryngeal, vulvar, or penile SCC. According to the literature, HPV6 is the most prevalent low-risk genotype and second most prevalent HPV genotype in penile SCC [35,40]. Interestingly, in our study, the dominant low-risk HPV was HPV42, which in three cases represented a single infection and in one case was associated with HPV6. In the other three cases positive for low-risk HPV, we found the presence of HPV82 (*n* = 1), HPV82 + HPV44 (*n* = 1), and HPV53 (*n* = 1). In five of seven tumors associated with low-risk HPV, the histological subtype was clearly HPV-associated with warty, basaloid, and warty–basaloid morphology, which supports HPV-induced carcinogenesis. The remaining two cases presented with high-grade tumors, which might have impaired the correct assessment of the histological type. Although low-risk HPV genotypes in our cohort may represent co-infection, low-risk HPV-driven carcinogenesis cannot be excluded. The case with HPV82 detected in both the primary tumor and its metastasis supports this possibility, though further molecular analyses are needed to clarify the underlying mechanisms.

HPV infection is responsible for a large number of SCCs in the oropharyngeal and anogenital areas. In patients with oropharyngeal tumors, HPV status has been shown to have a strong and independent prognostic significance regarding survival and response to radiation treatment [41]. As a result, HPV tumor status has been introduced as a criterion in the staging of oropharyngeal cancers in the most recent edition of the American Joint Committee on Cancer (AJCC 8th Ed) [42]. A comprehensive meta-analysis from 2017 demonstrated that patients with HPV-associated vulvar cancer exhibited improved survival rates compared to those with HPV-independent tumors [19]. Although less investigated, it appears that patients with HPV-associated penile cancer and positive lymph nodes have shown better survival when compared with patients with HPV-independent tumors [21]. No significant association was observed between HPV tumor status and the occurrence of ILNM within this dataset. This aligns with recent studies indicating that HPV tumor status does not influence the risk for ILNM [21].

In 78% (*n* = 36) of the HPV-positive SCC cases with ILNM, we discovered the same HPV genotype in the penile tumor and the ILNM, primarily high-risk HPV16. Of particular interest, HPV82 was present in both the penile tumor and metastasis in one of these cases. HPV82 is seldom found in invasive penile cancer and, in the literature, it appears only as an uncommon finding [43]. To our knowledge, no cases of metastatic HPV82-positive invasive penile cancer have been reported. Seven HPV-positive SCC cases had HPV-negative metastases. We found a very faint HPV signal in two of these cases, indicating a low viral load in the primary tumor. In two other discordant cases, we discovered that the penile tumor had both low-risk and high-risk genotypes: HPV16 and HPV42 in one case and HPV16 and HPV6 in another. Only the high-risk HPV16 genotype was found in the metastases in both cases, potentially indicating the high-risk genotype as the driver mutation.

Penile SCC includes a variety of histological subtypes with distinct morphologies, histological grades, growth patterns, and different risks for ILNM. According to the EAU, the subtypes associated with good prognosis and low metastatic risk are verrucous subtype with its more locally destructive variant carcinoma cuniculatum, the pseudohyperplastic variant of usual subtype, and papillary and warty subtypes. The usual subtype and most mixed forms as well as the pleomorphic variant of warty subtype have been associated with an intermediate risk for lymph node metastasis. In the high-risk category, we find sarcomatoid, basaloid, and warty–basaloid, as well as tumors with a high tumor grade [44]. Our data on histological subtype and metastatic risk confirm a low risk in verrucous penile carcinoma with no ILNM in the 17 included cases. We found a high metastatic rate in cases with usual, basaloid, warty, and warty–basaloid subtypes. Our findings suggest a higher metastatic rate in the warty subtype of squamous cell carcinoma than the 10–18% reported in the literature [45,46]; however, these results should be interpreted with caution given the limited sample size.

According to the WHO, penile cancer should be classified into HPV-associated and HPV-independent histological subtypes. Since molecular testing of tumor HPV status is mostly used in research settings, p16 immunohistochemical staining serves as a proxy for the presence of high-risk HPV genotypes. Our work revealed a very good concordance between p16 immunohistochemical staining and HPV tumor status, with a Cohen’s κ of 0.83 (91.6% agreement). In countries with lower resourced laboratory settings, penile tumors may be classified into HPV-associated and HPV-independent subtypes based on morphology alone. Our results support this claim, demonstrating a good concordance between the histological subtype of SCC and HPV tumor status (PCR result), evidenced by a Cohen’s kappa (κ) of 0.80, reflecting a 90.7% agreement. For our dataset, no significant association was observed between p16 immunohistochemical staining and ILMN.

Some studies have shown that patients with penile tumors associated with TP53 mutations showed a higher risk for ILNM [47,48], as well as a significantly lower survival rate compared with patients with tumors negative for TP53 mutations [24]. For our dataset, no significant association was observed between p53 immunohistochemical staining and ILMN. Importantly, in this study, the addition of HPV status, p16 expression, and p53 expression did not significantly improve the multivariable model beyond the established clinicopathological factors. Although these biomarkers are frequently proposed as prognostic indicators in penile cancer, our findings suggest that their incremental predictive value for ILNM may be limited when key pathological features such as lymphovascular invasion, perineural invasion, and tumor grade are already accounted for. Importantly, the lack of statistical significance for HPV, p16, and p53 in our model should not necessarily be interpreted as the absence of biological relevance but, rather, as limited additional predictive utility in the multivariable clinical context. These findings underscore the importance of evaluating biomarkers within fully adjusted models rather than in isolation.

Notably, p53 expression was also not associated with ILNM in the HPV-negative subgroup, where p53 alterations might be expected to have higher biological relevance. This suggests that, even in HPV-negative disease, p53 expression provides limited additional clinical value once the established pathological risk factors have been adjusted for.

Our study has shown that, in most cases of HPV-positive penile cancer with ILNM, HPV-infected cells disseminate to the metastatic site. Our findings support a concordance between p16 immunohistochemical staining as well as histological subtype and HPV tumor status. Since neither HPV tumor status nor immunohistochemical expression of p16 and p53 was statistically significant between lymph node-positive and lymph node-negative patients with penile cancer in this dataset, further larger studies are required to identify factors associated with ILNM. Research utilizing genomic profiling in both HPV-associated and HPV-independent penile cancer may enhance the understanding of the metastatic process and facilitate personalized treatment for penile cancer patients. We acknowledge several limitations of the current study, including the lack of penile-specific validation of the TMA material used for immunohistochemical staining. Despite good concordance between whole tissue sections and TMA cores in other anogenital SCC, this may impact the interpretation of negative results. The absence of follow-up data on patient survival represents an additional limitation, as the present analysis is confined to evaluating the predictive, but not the prognostic, value of HPV tumor status and immunohistochemical expression of p16 and p53.

## 5. Conclusions

The overall HPV prevalence in this cohort was 42.9%, where 40.8% of the cases presented at least one high-risk HPV. Multiple low-risk HPV genotypes were also associated with penile SCC. In 80% of HPV-positive tumors with analyzable ILNM, the same HPV genotypes were identified in both the metastasis and invasive penile tumor. While not a perfect substitute for molecular HPV testing, IHC for p16 and morphological classification of SCC can serve as surrogates for molecular HPV testing in penile SCC in countries with limited laboratory resources. In this dataset, there was no significant association between HPV status, p16 expression, or p53 expression and the presence of ILNM, indicating a limited usefulness as predictive markers for ILNM in penile cancer. Consistently, in the multivariable analysis, the addition of HPV status, p16, and p53 did not improve the model performance beyond the established clinicopathological factors.

## Figures and Tables

**Table 1:** Selected patient and tumor characteristics.

	PeCa Cases (*n* = 343)	HPV-Positive (*n* = 147)	HPV-Negative (*n* = 196)	*p*-Value
Age at surgery, mean (sd)	69 (10.7)	70 (10.3)	69 (11.0)	0.55
pT stage, *n* (%)				0.76
Ta	2 (0.6)	1 (0.7)	1 (0.5)	
T1	129 (38.2)	58 (39.5)	71 (36.2)	
T2	137 (39.9)	54 (36.7)	83 (42.3)	
T3	75 (21.9)	34 (23.1)	41 (20.9)	
Grade, *n* (%)				<0.001
1	91 (26.5)	10 (6.8)	81 (41.3)	
2	146 (42.6)	80 (54.4)	66 (33.7)	
3	106 (30.9)	57 (38.8)	49 (25.0)	
pN stage, *n* (%)				0.83
pN−	203 (59.2)	86 (58.5)	117 (59.7)	
pN+	102 (29.7)	46 (31.3)	56 (28.6)	
pNx	38 (11.1)	15 (10.2)	23 (11.7)	

**Table 2:** HPV genotypes in the primary tumor and metastasis in patients with HPV-positive SCC.

	Primary Tumor Result	ILNM Result
Concordant results (*n* = 36)		
33	HPV16	HPV16
1	HPV18	HPV18
1	HPV35	HPV35
1	HPV82	HPV82
Discordant results (*n* = 9)		
1	HPV16 + HPV42	HPV16
1	HPV16 + HPV6	HPV16
4	HPV16	HPV-negative
1	HPV16 + HPV44	HPV-negative
1	HPV45	HPV-negative
1	HPV52	HPV-negative

**Table 3:** Histological subtypes and metastatic frequency.

Subtype	pN0	pN+	Total
Usual	79 (57.7%)	58 (42.3%)	137
Basaloid	25 (64.1%)	14 (35.9%)	39
Warty	11 (61.1%)	7 (38.9%)	18
Warty–basaloid	12 (54.5%)	10 (45.5%)	22
Verrucous	17 (100.0%)	0 (0%)	17
Mixed HPV-associated	29 (78.4%)	8 (21.6%)	37
Mixed HPV-independent	30 (85.7%)	5 (14.3%)	35

## Data Availability

Datasets are available from the corresponding author on reasonable request.
